# Molecular monitoring of *plasmodium falciparum *drug susceptibility at the time of the introduction of artemisinin-based combination therapy in Yaoundé, Cameroon: Implications for the future

**DOI:** 10.1186/1475-2875-11-113

**Published:** 2012-04-12

**Authors:** Sandie Menard, Isabelle Morlais, Rachida Tahar, Collins Sayang, Pembe Issamou Mayengue, Xavier Iriart, Françoise Benoit-Vical, Brigitte Lemen, Jean-François Magnaval, Parfait Awono-Ambene, Leonardo K Basco, Antoine Berry

**Affiliations:** 1 Service de Parasitologie-Mycologie, Centre Hospitalier Universitaire de Toulouse et UMR152 UPS-IRD, Université de Toulouse, Toulouse, France; 2 Institut de Recherche pour le Développement, UMR MIVEGEC, Montpellier, France; 3 Laboratoire de Recherche sur le Paludisme, Organisation de Coordination pour la lutte contre les Endémies en Afrique Centrale (OCEAC), Yaoundé, Cameroon; 4 Unité Mixte de Recherche 216, Institut de Recherche pour le Développement (IRD), Université Paris Descartes, Laboratoire de Parasitologie, Faculté de Pharmacie, Paris, France; 5 Centre de Formation et de Recherche en Médecine et Santé Tropicale, Faculté de Médecine Nord, Université de la Méditerranée, Marseille, France; 6 CNRS, LCC (Laboratoire de Chimie de Coordination), et Université de Toulouse Paul Sabatier, UPS, INPT, LCC, Toulouse, France; 7 Centre d'Animation Sociale et Sanitaire (CASS), Yaoundé, Cameroon; 8 CNRS UMR 5288 Anthropobiology, Université de Toulouse, Toulouse, France; 9 Unité Mixte de Recherche 198, Institut de Recherche pour le Développement (IRD), Faculté de Médecine La Timone, Université de la Méditerranée, Marseille, France

**Keywords:** Malaria, Cameroon, *pfcrt*, *pfmdr1*, *pfmdr1 *copy number, Resistance, LNA probes

## Abstract

**Background:**

Regular monitoring of the levels of anti-malarial resistance of *Plasmodium falciparum *is an essential policy to adapt therapy and improve malaria control. This monitoring can be facilitated by using molecular tools, which are easier to implement than the classical determination of the resistance phenotype. In Cameroon, chloroquine (CQ), previously the first-line therapy for uncomplicated malaria was officially withdrawn in 2002 and replaced initially by amodiaquine (AQ) monotherapy. Then, artemisinin-based combination therapy (ACT), notably artesunate-amodiaquine (AS-AQ) or artemether-lumefantrine (AL), was gradually introduced in 2004. This situation raised the question of the evolution of *P. falciparum *resistance molecular markers in Yaoundé, a highly urbanized Cameroonian city.

**Methods:**

The genotype of *pfcrt *72 and 76 and *pfmdr1 *86 alleles and *pfmdr1 *copy number were determined using real-time PCR in 447 *P. falciparum *samples collected between 2005 and 2009.

**Results:**

This study showed a high prevalence of parasites with mutant *pfcrt *76 (83%) and *pfmdr1 *86 (93%) codons. On the contrary, no mutations in the *pfcrt *72 codon and no samples with duplication of the *pfmdr1 *gene were observed.

**Conclusion:**

The high prevalence of mutant *pfcrt *76T and *pfmdr1 *86Y alleles might be due to the choice of alternative drugs (AQ and AS-AQ) known to select such genotypes. Mutant *pfcrt *72 codon was not detected despite the prolonged use of AQ either as monotherapy or combined with artesunate. The absence of *pfmdr1 *multicopies suggests that AL would still remain efficient. The limited use of mefloquine or the predominance of mutant *pfmdr1 *86Y codon could explain the lack of *pfmdr1 *amplification. Indeed, this mutant codon is rarely associated with duplication of *pfmdr1 *gene. In Cameroon, the changes of therapeutic strategies and the simultaneous use of several formulations of ACT or other anti-malarials that are not officially recommended result in a complex selective pressure, rendering the prediction of the evolution of *P. falciparum *resistance difficult. This public health problem should lead to increased vigilance and regular monitoring.

## Background

Monitoring the level of *Plasmodium falciparum *resistance against anti-malarial drugs is one of the keys to a successful malaria control. Although controlled clinical trials are the best available tool for assessing the relevance of anti-malarial treatments, molecular monitoring offers some advantages. Studies on single-nucleotide polymorphisms (SNPs) and duplication of genes involved in resistance can be carried out with more ease and are less time-consuming. Furthermore, molecular monitoring may reveal trends, allowing anticipation in the changes of treatment policies.

In Cameroon, the first-line recommended therapy for uncomplicated malaria was chloroquine (CQ) until 2002 and amodiaquine (AQ) monotherapy between 2002 and 2004. In January 2004, the artesunate-amodiaquine (AS-AQ) combination was officially adopted and artemether-lumefantrine (AL) was added as an alternative artemisinin-based combination therapy (ACT) in 2006 [1]. In practice, AS-AQ and AL have been used nationwide since 2007. AS-AQ is widely available in public health care centres while AL is relatively less prescribed because of its low supply in the public sector at a subsidized price [2].

The amplification of *pfmdr1 *gene is a common molecular marker of mefloquine (MQ) resistance. An increase in the *pfmdr1 *copy number is associated with clinical failures to MQ [3] and *in vitro *resistance to lumefantrine, which is an amino-alcohol, like MQ [4]. The amplification of *pfmdr1 *gene has also been demonstrated to decrease the susceptibility to artemisinin derivatives in the field as well as in laboratory-adapted *P. falciparum *strains [4-8]. Furthermore, a recent study in eastern Sudan reported an association between the carriage of parasites with increased *pfmdr1 *copy number before treatment and recurrent parasitaemia after AL therapy [9].

*In vitro*, the *pfmdr1 *N86 wild-type allele, independently of the *pfmdr1 *copy number, is associated with a higher susceptibility to lumefantrine and MQ [3,10,11]. In parallel, in the field, *pfmdr1 *N86 and *pfcrt *K76 wild-type alleles were selected by artemether-lumefantrine (AL) treatment whereas they were not selected by artesunate-amodiaquine (AS-AQ) or amodiaquine-sulphadoxine-pyrimethamine (AQ-SP) [12-15]. Conversely, the *pfmdr1 *86Y and *pfcrt *76T mutant alleles are associated with CQ resistance and also with AQ monotherapy failure [16-19]. Likewise, these haplotypes are selected by the association AS-AQ [20,21].

A *pfcrt *genotype conferring high levels of resistance to AQ, corresponding to SVMNT haplotype of the codons 72-76, has been identified, first in Tanzania and more recently in Angola [22,23]. This haplotype, widely observed in Asia and South America, seems to be strongly selected by the use of AQ [24,25].

The objective of this study was to determine the prevalence of *pfmdr1 *multiple copies and mutant *pfcrt *72 and 76 and *pfmdr1 *86 codons in Yaoundé, Cameroon at the time of the introduction of ACT. It is important to have a "molecular snapshot" of *P. falciparum *isolates at the beginning of this new anti-malarial therapeutic strategy, first, in order to make meaningful comparisons in the future and, secondly, to determine if there is any evidence of molecular mark suggesting a rapid evolution towards resistance.

## Methods

### Study sites and origin of samples

The study was carried out between 2005 and 2009, on a total of 447 samples from patients with a microscopy-confirmed diagnosis of uncomplicated *falciparum *malaria. The recruitment sites were in Yaoundé *intra-muros *(3° 52' N, 11° 31' E), including the healthcare centre of Nkolndongo (49 patients, median of three years old [0 month to 47 years]), the healthcare centre of Olembe (42 children, median of 2.5 years old [eight months to 12 years]), and the healthcare centre of Nlongkak (125 patients, median of two years old [six months to five years]). Two hundred and thirty-one samples were obtained from asymptomatic children aged from five to 11 years in Mfou (3°43'N, 11°38'E), 26 km from the centre of Yaoundé. This study was reviewed and approved by the Cameroonian National Ethics Committee and Cameroonian Ministry of Public Health.

Before patients with a positive thick smear have received an ACT treatment, finger-pricked capillary blood sample was collected on different filter papers, Whatman (Whatman plc, Middlesex, UK) or IsoCode STIX (Schleicher & Schuell, Keene, NH, USA). DNA from paper filters was extracted using the chelex-100 boiling method [26], concentrated by ethanol precipitation and frozen at -20°C until amplification.

### Determination of *pfmdr1 *copy number

To determine the copy number of *pfmdr1*, a qPCR method described previously was used [27]. All samples were tested in triplicate in 96-well plates on a LightCycler^® ^480 system (Roche Diagnostics, Neuilly sur Seine, France). Each run included two control DNA samples of reference *P. falciparum *clones, FCM29/Cameroon and Dd2/Indochina, which are known to have one and two-three copies of *pfmdr1 *gene, respectively [27].

### Genotyping of *pfcrt *and *pfmdr1*

Genotyping of *pfcrt *76 and *pfmdr1 *86 codons was performed with a qPCR assay using Fluorescence Resonance Energy Transfer (FRET) hybridization probes and an analysis of the melting curve described previously [28,29]. Each run included two control DNA samples of *P. falciparum*: the CQ-susceptible F32/Tanzania strain with *pfcrt *K76 and *pfmdr1 *N86 wild-type alleles and the CQ-resistant FCM29/Cameroon clone, carrying *pfcrt *76T and *pfmdr1 *86Y mutant alleles.

The detection of the *pfcrt *72S mutant allele was performed with a TaqMan probe-based genotyping assay the originality of which resides in the presence of Locked Nucleic Acid (LNA) inside the probes. LNA is a synthetic RNA analogue which, when integrated into an oligonucleotide, shows a strong affinity for their complementary targets [30]. Because of their thermal stability when hybridized to DNA, oligonucleotides containing LNA have a higher melting temperature (T_m_) and could be used as primers, probes or clamps to improve molecular detection [31-33]. In general, sequences from *P. falciparum *contain a high percentage of adenine (A) and thymine (T) resulting in a low Tm and complicating molecular analysis. The introduction of LNA bases is a powerful tool to obtain discriminating probes with a moderate length and a probe hybridization that may occur during the annealing step in PCR. Consequently, this technique was particularly well suited for the experimental conditions described here. The *pfcrt *gene was amplified by using primers P.falcA (5'-CAATT TTgTTTAAAgTTCTTTTAgCAA-3') and P.falcF (5'-gTTCTTgTCTTggTAAATgTgCTCA-3'). To genotype the different alleles, the amplified product was detected with one of the specific TaqMan probes: AF233067 probe, 5'-YAK-AATTgTATTCATT + A + C + ACTT + A + CA--BBQ-3' hybridized with the *pfcrt *72S mutant allele (SVMNT haplotype) and HM854027 probe, 5'-LC670-AATTgTTTCAATT + A + C + ACATA + CA--BBQ-3' hybridized with the *pfcrt *C72 wild-type allele (CVIET haplotype) (the presence of a LNA nucleotide is preceded by the sign +). The primers and probes were designed in collaboration with Tib MolBiol Syntheselabor (Berlin, Germany). Master mixes contained 1 μl GeneAmp^® ^10 × PCR Gold Buffer (Applied Biosystems, Branchburg, NJ), 2.5 mM MgCl_2_, 200 μM pooled dNTP, 500 nM of forward and reverse primers, 250 nM of each probe, 1 U per reaction of AmpliTaq^® ^Gold DNA Polymerase (Applied Biosystems) and 2 μl of template DNA for a total reaction volume of 10 μl. Each reaction plate was run with control DNA samples of *P. falciparum*, in particular the 7G8/Brazil strain known to harbour the *pfcrt *72S mutated allele [34], F32/Tanzania and FCM29/Cameroon as *pfcrt *C72 wild-type control [28], water and DNA of healthy patient, which served as negative external amplification controls. The multiplex TaqMan assay reactions were carried out in a LightCycler^® ^480 Multiwell Plate 384 (Roche Diagnostics) with the following PCR programme: an initial step at 95°C for 12 minutes followed by 45 cycles of 10 seconds at 95°C and 45 seconds at 60°C. Data analysis for allelic discrimination was performed with the LightCycler^® ^480 software (Roche Diagnostics).

### Statistical analysis

The proportions were compared using χ^2 ^test thanks to SigmaStat^® ^software. The significance level (*P*) was fixed at 0.05.

## Results

### *Pfmdr1 *copy number

The copy number of *pfmdr1 *was determined for only 215 isolates from healthcare centres of central Yaoundé because of the limited amount of DNA samples from Mfou. Regardless of where the tested isolates were collected, none of them were identified with *pfmdr1 *gene amplification (Table 1). The estimated gene copy number from all analysed isolates was close to 1, with an average copy number of 1.05 and a standard deviation of 0.20 (data not shown).

**Table 1:** Frequency of mutations and/or gene amplification in anti-malarial resistance markers *pfmdr1 *and *pfcrt *in *Plasmodium falciparum *isolates in Yaoundé and Mfou, Cameroon

Genes and alleles	Number of samples (%)						P
	**Both sites**		**Yaoundé**		**Mfou** ***(Suburb of Yaoundé)***		

***pfmdr1 *amplification **(n^a ^= 215, n^b ^= NA)							

1 *pfmdr1 *copy number	215	(100%)	215	(100%)	NA	NA	NA
	
> 1 *pfmdr1 *copy number	0	(0%)	0	(0%)	NA	NA	

***pfmdr1 *codon 86 **(n^a ^= 201, n^b ^= 209)							

Mutant 86Y haplotype only	328	(80%)	153	(76%)	175	(84%)	NS
	
Mixed N86 and 86Y haplotypes	53	(13%)	28	(14%)	25	(12%)	
	
Wild-type N86 haplotype only	29	(7%)	20	(10%)	9	(4%)	

**Total mutant 86Y haplotype**^**c**^	**381**	**(93%)**	**181**	**(90%)**	**200**	**(96%)**	**0.042**^**d**^

***pf*c*rt *codon 76 **(n^a ^= 203, n^b ^= 229)							

Mutant 76T haplotype only	270	(62%)	145	(71%)	125	(55%)	< 0.001^e^
	
Mixed K76 and 76T haplotypes	89	(21%)	20	(10%)	69	(30%)	
	
Wild-type K76 haplotype only	73	(17%)	38	(19%)	35	(15%)	

**Total mutant **76T **haplotype**^**c**^	**359**	**(83%)**	**165**	**(81%)**	**194**	**(85%)**	**NS**^**d**^

***pf*c*rt *codon 72 **(n^a ^= 202, n^b ^= 212)							

Mutant 72S haplotype	0	(0%)	0	(0%)	0	(0%)	NS
	
Wild-type C72 haplotype	414	(100%)	202	(100%)	212	(100%)	

NA: not analysable because of the limited amount of DNA samples

NS: non significant

^a^: Number of total samples tested in the centre of Yaoundé

^b^: Number of total samples tested in the suburb of Yaoundé

^c^: Total mutant haplotype regroups single mutant haplotype and mixed haplotype, all samples with mixed genotype for the considered allele are classified in mutant group.

^d^: comparison of total mutant haplotypes *versus *wild-type haplotypes

^e^: comparison of mutant, wild-type and mixed haplotypes

### *Pfmdr1 *and *pfcrt *genotypes

The prevalence of *pfmdr1 *and *pfcrt *alleles in blood samples obtained from different sites in Yaoundé is presented in Table 1. The frequencies of the *pfmdr1 *86Y mutant genotype were 76% (153/201) and 84% (175/209) in Yaoundé and Mfou, respectively. Wild-type *pfmdr1 *N86 genotype was observed in 10% (20/201) and 4% (9/209) of isolates, and 14% (28/201) and 12% (25/209) of isolates presented a mixed genotype in Yaoundé and Mfou, respectively. No significant differences were observed between Yaoundé and Mfou.

The frequencies of *pfcrt *76T mutant genotype were 71% (145/203) and 55% (125/229), the *pfcrt *K76 wild-type allele was present in 19% (38/203) and 15% (35/229) and mixed *pfcrt *alleles occurred in 10% (20/203) and 30% (69/229) of the isolates in Yaoundé and Mfou, respectively, with a significant difference (p < 0.001).

Contrary to *pfcrt *76, a significant difference (p = 0.042) of the distribution of the alleles was observed between Yaoundé and Mfou when all samples with mixed *pfmdr1 *86 genotype are classified in mutant group.

No significant differences were observed either between the different healthcare centres of Yaoundé or between the different times of sample collection (data not shown).

None of the 414 samples tested for the codon 72 of *pfcrt *gene was found with the mutant 72S allele (SVMNT haplotype).

## Discussion

As elsewhere in the world, a very rapid development of resistance to anti-malarial drugs in Africa requires a regular monitoring in multiple and strategic points. With 53% of the population living in cities against 38% WHO African region, Cameroon is a highly urbanized African country [35]. This demonstrates the importance of epidemiological studies in large cities such as Yaoundé, which currently has a population of over 1,800,000 inhabitants.

In the present study, a high prevalence of mutations associated with drug resistance was found in Yaoundé and its suburbs in both codon 76 of the *pfcrt *gene (83%) and codon 86 of the *pfmdr1 *gene (93%) when all samples with mixed genotype were classified as mutant (Table 1). These results are in agreement with several other studies. Previous works of Basco *et al *carried out in Yaoundé showed that 70% of 157 *P. falciparum *clinical isolates had the mutant *pfcrt *76T codon in 2001 [36], and a large majority of isolates (88% of 64) carried the *pfmdr1 *86Y mutant allele between 1997 and 2000 [37]. Similarly, Mbacham *et al *reported 77% and 76% prevalence of mutant *pfcrt *76T and *pfmdr1 *86Y codons, respectively, in samples collected during the period 2004-2006 in Yaoundé [38]. Despite different classification of double populations and techniques with different sensitivity, the prevalence of mutations appears to increase (*pfcrt*) or remains at a high and relatively stable level (*pfmdr1*) until 2009 in spite of the official withdrawal of CQ from Cameroon in 2002. In some endemic areas, stopping the widespread use of CQ resulted in a return of chloroquine-sensitivity associated with the reappearance of wild-type genotypes. In the absence of drug pressure, *P. falciparum *wild-type haplotypes have a selective advantage over mutants. For example, in 1993, Malawi was the first sub-Saharan African country to replace CQ with SP nationwide in response to the high rates of CQ-resistant *falciparum *malaria. This change resulted in a decrease in the prevalence of the mutant *pfcrt *haplotype associated with CQ resistance from 85% in 1992 to 13% in 2000. For *pfmdr1 *86Y mutant codon, the same study showed similar results but with lower amplitude (from about 60% in 1993 to around 20% in 2000) [39]. The recovery of CQ-sensitivity phenotype and genotype was also observed elsewhere in Malawi [40], Kenya [41] and China [42].

This stability of mutant *pfcrt *76T and *pfmdr1 *86Y genotypes observed in Yaoundé and suburb may be the result of many factors. First of all, the choice of the replacement treatment logically influences the type of selected isolates. The use of SP, which has no influence on the selection of mutant *pfcrt *and *pfmdr1 *genotypes, has been shown to favour, by a phenomenon of selective advantage, the reappearance of CQ-sensitive isolates harbouring wild-type *pfcrt *K76 and *pfmdr1 *N86 genotypes [39-41]. The use of AL or artesunate-mefloquine (AS-MQ) seems to favour the return to the predominance of wild-type *pfmdr1 *N86 genotype and, to a lesser extent, to the wild-type *pfcrt *K76 genotype by an active selection [14,43-45]. Inversely, AQ, a close Mannich base analogue of CQ, or AS-AQ promotes the maintenance of CQ-resistant isolates with the mutant *pfcrt *and *pfmdr1 *genotypes by an active selective pressure [20,46], as observed in the present study. Whereas in East African countries like Malawi or Kenya, SP or AL had largely replaced CQ [47], in Yaoundé, in 2005, AQ was still prescribed as a first-line anti-malarial drug in 20% and 63% of adults and children under five years old, respectively [2], and AS-AQ in 4.5% and 1.5%. AL was used only in 8.3% and 2.4%, AS-MQ in 1.5% and 0.8%, and SP in 5.8% and 0% of adults and children less than five years old, respectively [2].

Secondly, the changes of *P. falciparum *resistance phenotype and genotype after the withdrawal of CQ depend on the rapidity of drug replacement. For example, in Malawi where a profound and rapid return to CQ sensibility was observed, the change in drug policy from CQ to SP was swift and efficient, so that SP became the only available anti-malarial drug in less than one year after the implementation of the new drug policy. In contrast, these changes were progressive and lasted several years in many areas as in Cameroon. In fact, in Yaoundé, although the National Malaria Control Programme of Cameroon had replaced CQ by AQ in 2002 and then AQ monotherapy by AS-AQ since January 2004, CQ was still largely accessible through the informal outlets (e.g. food market) in August 2005 [2].

Finally, in a more general way, fitness loss of mutant *P. falciparum *might be associated with the development of compensatory mechanisms able to maintain mutant parasites even in the absence of drug pressure [48]. This feature might explain, at least in part, the persistence of mutant *pfcrt *codon in Southeast Asia and South America [49-51] and also in Cameroon, as described here.

In Mfou, a higher frequency of mixed *pfcrt *haplotypes was observed at the expense of mutant *pfcrt *population. This observation was not done for *pfmdr1 *haplotypes. A possible reason for this observation is a drug pressure selection different from that existing in Yaoundé.

Since the probes used to detect the mutation in codon 76 of *pfcrt *gene were not able to detect that of codon 72, a new technique using LNA probes was developed in the present study to discriminate the mutant SVMNT haplotype (72S mutation) from the wild-type CVIET haplotype (C72 wild-type). Previous studies and data collected from countries like Bolivia or India suggested that AQ has an early and prominent role in the selection of parasites carrying SVMNT haplotype associated with drug resistance [24]. These parasites are highly resistant to AQ, but only moderately resistant to CQ. Contrary to CVIET haplotype, once the SVMNT haplotype emerges in a given parasite population and CQ and AQ are removed, the repopulation of sensitive strains may be very slow to occur [24]. As the SVMNT haplotype was recently described in Tanzania and Angola [22,23], it was important to verify whether this haplotype existed in Yaoundé. None of the samples tested for the codon 72 of *pfcrt *was found to carry the SVMNT haplotype. These results are contrary to what was observed in nearby African countries, such as in Ghana [52], Tanzania [22] and Angola [23] where the prevalence of this haplotype was between 3.9% and over 50%. It is possible that the observed predominance of SVMNT haplotype in Angola is the result of frequent travels of Brazilian and Angolan citizens between the two countries [23], which is not the case in Cameroon. However, the monitoring of *pfcrt *codons 72-76 should be pursued because AQ has long been prescribed in Cameroon before and since the cessation of the use of CQ (2002) and until 2005 [2] and seems to have an important role in the selection of the SVMNT haplotype [22].

The amplification of *pfmdr1 *gene has been more closely linked to MQ and halofantrine (HAL) resistance [53-55]. In this study, *pfmdr1 *amplification was not observed in Yaoundé between 2005 and 2009. Elsewhere in Africa, the situation seems to be contrasted. In various studies conducted in East Africa, only four samples were found with *pfmdr1 *gene duplication, one in Kenya and three in Sudan (near the Ethiopian border) among 475 isolates tested (57 in Sudan [9], 72 in Kenya [46], 186 in Zanzibar [53] and 160 in Malawi [54]). In West Africa, on the one hand, none of 580 samples tested in Liberia and Guinea-Bissau between 1981 and 2005 was found to be duplicated [55]; on the other hand, two studies had identified in Burkina Faso, Ivory Coast, Togo and Madagascar, six *pfmdr1 *duplications among 112 samples tested [27,56]. In Central Africa, data are limited since only 32 samples were screened and all of them had a single copy of *pfmdr*1 gene [27]. In this region, an exception is the study of Uhlemann *et al *who found the duplication of *pfmdr1 *gene in five of 62 clinical isolates tested (8%) in 1995 in Lambaréné, Gabon [57]. Four of these five patients harboured the wild-type N86 *pfmdr1 *codon even though during this period 90% of isolates carried the mutant *pfmdr1 *codon 86 around Lambaréné [58]. However, in 2002 at the same study site, none of 37 samples tested had *pfmdr1 *gene duplication. These observations on *pfmdr1 *gene amplification in Lambaréné are difficult to explain outside of the possible selection of such a clone by previous clinical trials on the same site, using low dose of mefloquine [58-60]. Nevertheless, these data showed that *P. falciparum *isolates from Central Africa can have *pfmdr1 *gene duplication.

The lack of *pfmdr1 *gene duplication in Yaoundé may possibly be due to the very low use of MQ or HAL, which represented only 1.5% of first-line treatments against malaria in 2005 [2], but also partly to the high prevalence of the *pfmdr1 *Y86 mutant allele. Indeed, in Southeast Asia, *pfmdr1 *amplification has been suggested to be incompatible in the presence of the mutant *pfmdr1 *86Y allele [61]. However, the conclusion of that Asian study has not been confirmed in Africa, where the existence of parasites harbouring a duplicated *pfmdr1 *gene with mutant 86Y codon has been reported from Sudan [9], Gabon [57] and Ivory Coast [27,56].

The molecular analysis performed in the present study did not find any *pfcrt *72S mutation, which may be a good sign for the continued use of AQ in combination with AS. A regular evaluation of AS-AQ efficacy, in parallel with molecular surveillance, is required to ensure the utility of AS-AQ in Cameroon. This ACT contributes to the maintenance of a high prevalence of mutant *pfcrt *76T and *pfmdr1 *86Y alleles. The pressing question is to predict how these parasites will evolve in the presence of AL pressure. Several scenarios can be envisioned. Firstly, they could behave like Southeast Asian isolates and will not progress to the duplication of *pfmdr1 *gene in the absence of wild-type *pfmdr1 *N86 allele. Secondly, as already observed in some cases in Africa [9,27,56,57], the parasites may acquire multicopies of *pfmdr1 *despite the *pfmdr*1 86Y mutation. Only regular and exhaustive molecular monitoring of *P. falciparum *clinical isolates can provide the answer. However, the relevance of these results would be improved if they were associated with information on different anti-malarial drugs that are really taken by the patients because these data often differ from the current national recommendation.

## Abbreviations

ACT: Artemisinin-based combination therapy; AL: Artemether-lumefantrine; AQ: Amodiaquine; AQ-SP: Amodiaquine-sulphadoxine-pyrimethamine; AS: Artesunate; AS-AQ: Artesunate-amodiaquine; AS-MQ: Artesunate-mefloquine; CQ: Chloroquine; FRET: Fluorescence resonance energy transfer; HAL: Halofantrine; LNA: Locked nucleic acid; MQ: Mefloquine; *pfcrt*: *Plasmodium falciparum *chloroquine resistance transporter; *pfmdr1*: *Plasmodium falciparum *multidrug resistance gene 1; qPCR: Quantitative polymerase chain reaction; SNP: Single-nucleotide polymorphism; SP: Sulphadoxine-pyrimethamine; Tm: Melting temperature.

## Competing interests

The authors declare that they have no competing interests.

## Authors' contributions

SM, PIM, carried out the molecular genetic studies. SM, AB, XI conceived and designed the study protocol and analysed the results. SM, AB, FBV, LKB drafted the manuscript. JFM, FBV, XI participated in its design and helped to draft the manuscript. RT, CS, LKB and BL participated in study design, supervised clinical and laboratory diagnosis in the health care centres, and collected blood samples in Nlongkak, Olembe, and Nkolndongo, IM and PAA had the same role for the blood samples collected in Mfou. All authors read and approved the final manuscript.
